# Paracetamol or ibuprofen? A pilot study comparing rescue therapy for PDA in preterm infants within the first month

**DOI:** 10.3389/fped.2025.1717284

**Published:** 2026-01-02

**Authors:** Arindam Mukherjee, Anupam Gupta, Catherine Fullwood, Ruth Gottstein

**Affiliations:** 1 The University of Manchester, Manchester, United Kingdom; 2 Manchester University NHS Foundation Trust, Manchester, United Kingdom

**Keywords:** ibuprofen, neonates, paracetamol (acetaminophen), patent ductus arteriosus (PDA), prematurity, randomised trial, side effect

## Abstract

**Aims and objectives:**

To evaluate a study design aimed to assess the effectiveness and safety of IV paracetamol compared to IV ibuprofen for rescue treatment of clinically and hsPDA in preterm infants.

**Setting:**

This pilot randomised controlled trial was conducted in the regional neonatal intensive care unit in Manchester, UK, between September 2021 and August 2023.

**Patients and methods:**

The study recruited preterm infants (gestational age <32 weeks or birth weight <1,500 g) diagnosed with hsPDA via echocardiogram and presenting with signs and symptoms of PDA. Participants were randomised to receive either paracetamol or ibuprofen within the first 28 days of life. The primary outcome was closure or reduction to non-hsPDA, while secondary outcomes included the incidence of short- or medium-term complications of prematurity, such as BPD, NEC, IVH, and ROP. Adverse effects of medications were also reviewed to ensure safety. As part of the pilot design, recruitment, retention and data completeness were evaluated.

**Results:**

A total of 32 infants were recruited within a 2-year study period. Overall, both groups of infants showed similar baseline characteristics, although ibuprofen group had slightly smaller and sicker infants, despite randomisation. A high proportion of eligible babies were recruited (91.4%) and completed the trial. Post-intervention, 37.5% (16.1–50.0%) of infants in the paracetamol group converted to non-hsPDA, compared to 25.0% (7.3–52.4%) in the ibuprofen group (*p* = 0.704). There were no statistically significant differences between the two groups in short- or medium-term complications of prematurity or adverse effects. The trial also demonstrated feasibility by achieving the desired sample size in the given time frame with 91% consent and 100% completion.

**Conclusions:**

The PAIR (Paracetamol and Ibuprofen Research) trial indicated no differences in efficacy or safety between the two treatments. A larger study is required to validate the findings; this trial has demonstrated the feasibility of such a study.

**Clinical Trial Registration:**

## Introduction

The management of PDA is debated, but clinicians often use medication to treat symptomatic infants with hemodynamically significant PDA (hsPDA) (1, 2). In the UK, ibuprofen is the standard treatment, but studies suggest paracetamol may be a viable alternative (3, 4). A 2020 survey showed that 82% of level 3 NICUs in the UK use off-label paracetamol, with two-thirds (65%) using it as a second line and 10% using it as a first-line treatment (4). However, many neonatal units (47%) were unsure about its effectiveness (4). National surveys have shown that the dosages, treatment duration, and monitoring practices for infants using paracetamol vary significantly among NICUs in the UK (3, 4). There is a strong interest among neonatologists in the UK in participating in a randomised controlled trial (RCT) to compare paracetamol and ibuprofen to determine the most effective pharmacotherapy for managing hsPDA (4).

Evidence available from systematic reviews does not support routine prophylactic treatment for PDA within 24 h or early selective treatment within 72 h (2, 5). The rate of spontaneous closure of PDA in infants <28 weeks of gestational age is low (2). While most premature infants will likely see the PDA resolve on its own, a small number may develop a significant PDA that requires rescue treatment. There are no RCTs that have compared the effectiveness of intravenous paracetamol to IV ibuprofen for rescue treatment of hsPDA beyond two weeks of life (1, 2). The American Academy of Paediatrics (AAP) recently published a clinical report (April 2025) which recommends consideration of pharmacologic closure of PDA after two weeks despite a lack of evidence (2). This is because, if medical management fails, invasive interventions such as PDA ligation or medical device closure may become necessary.

The PAIR trial aimed to investigate rescue treatment for clinically and hemodynamically significant PDA. This trial was conducted as a prospective, randomised controlled, single-centre pilot study to evaluate the safety and efficacy of IV paracetamol compared to IV ibuprofen in a small group of extremely premature infants with hsPDA during the first four weeks of life. The findings from this small-scale study are anticipated to contribute to the existing evidence base and help inform a larger multi-centre trial aimed at guiding clinical practice.

### Study approval and trial registration

The study was approved by the Health Research Authority (HRA). The Research Ethics Committee (REC) and MHRA approved the CWOW application system (Reference ID 21/EE/0085, EudraCT number 2020-003863-25). A peer and PPI (Patient and Public Involvement) review was conducted, and the study was sponsored by Manchester University NHS Foundation Trust.

## Methods and data collection

This single-centre, prospective pilot randomised controlled study aimed to evaluate a study to assess the effectiveness and safety of paracetamol compared to ibuprofen in treating symptomatic preterm infants with hsPDA during the first four weeks of life. Based on a review of available resources, the recruitment strategy and timeline, a pragmatic sample size of 32 participants was determined for the pilot trial to evaluate specific outcomes over the two-year study period. The sample size was a pragmatic, feasibility-based estimate informed by expected recruitment rates, resource constraints, and single-centre practical considerations.

Inclusion criteria were gestational age <32 weeks or birth weight <1,500 g, with a postnatal age of ≤28 days, and babies meeting the criteria for the diagnosis of hsPDA on echocardiogram and deemed to be clinically symptomatic by the attending clinician. Infants were considered symptomatic if they exhibited signs and symptoms of a significant PDA and the clinician recognised that the PDA was adversely influencing the baby's clinical status, prompting the need for treatment.

**Figure 1:**
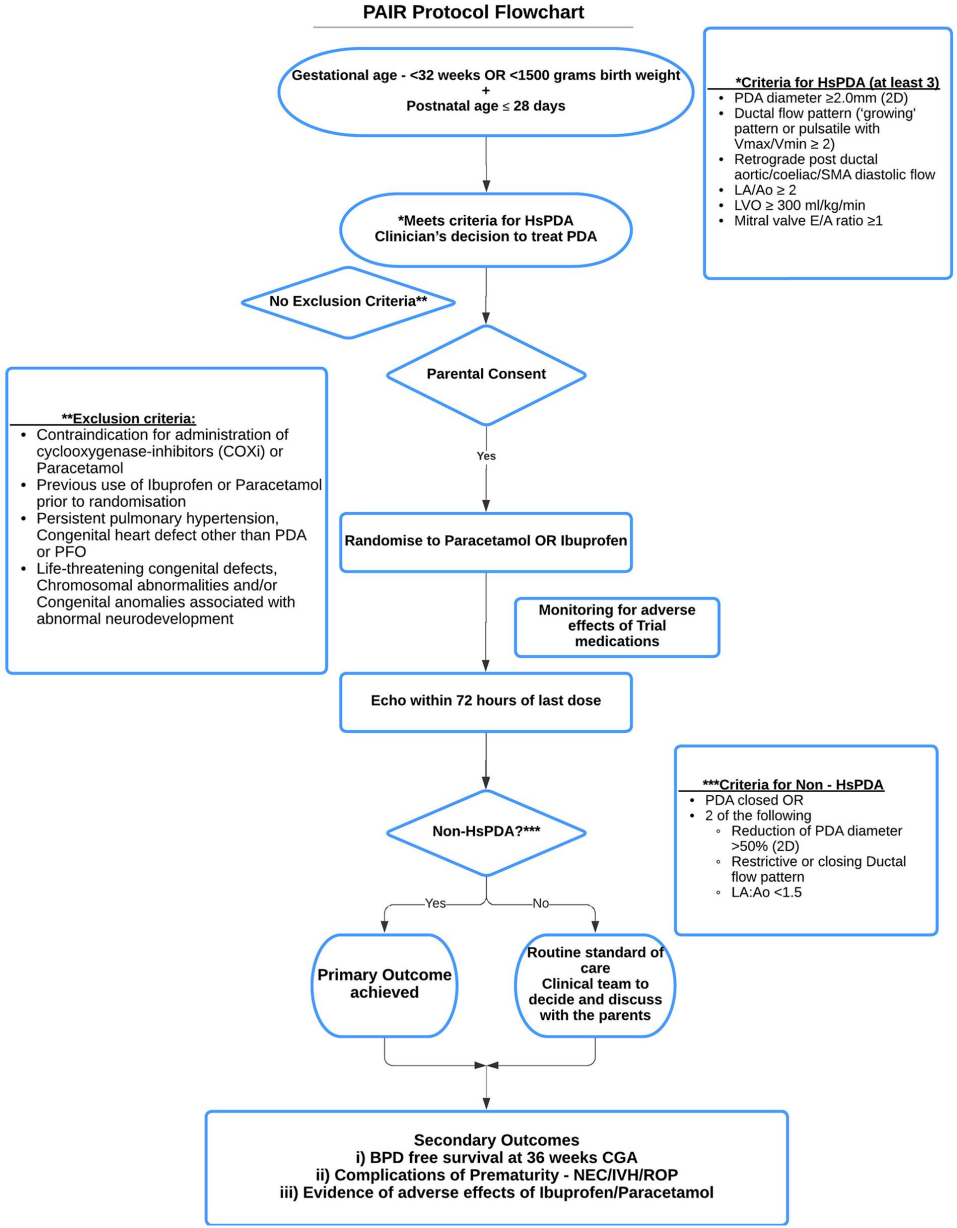
Trial summary flowchart for the PAIR trial.

The study defined hsPDA and non-hsPDA, as illustrated in Figure 1. Echocardiogram determinants were adapted from the European NPE (Neonatologist Performed Echocardiography) specialist interest group (6). HsPDA were defined by the presence of at least three of the following six criteria: PDA diameter of 2 mm or greater (2D), a ductal Doppler flow pattern which was either growing or pulsatile (with a Vmax/Vmin ratio 2 or greater), retrograde flow observed in postductal aortic, celiac, or superior mesenteric artery diastolic flow, a left atrial to aortic diameter ratio of 2 or greater, left ventricular output of 300 mL/kg/min or greater, and a mitral valve E/A ratio 1 or greater. Non-hsPDA were defined as a closed PDA or any two of the following three criteria: reduction in the PDA diameter by 50% in 2D, restrictive or closing ductal Doppler flow pattern, a left atrial to aortic diameter ratio of less than 1.5. The decision to treat infants with hsPDA rested with the responsible clinician. All screening echocardiograms for hsPDA were performed by one of three designated trial investigators (on delegation log) who were trained in performing neonatal echocardiograms to reduce interobserver variability. Once a decision to treat PDA was taken, parents were approached for consent to participate in the trial. Infants were randomly assigned to either arm using the website sealedenvelope.com. A repeat echocardiogram was performed within 72 h after treatment completion to reevaluate the status of the PDA. Blinded reporting of all echocardiograms by three trial- trained designated paediatric cardiologists was used to minimise bias and ensure reliability. After achieving the primary outcome and completing the trial medications, open-label treatment was allowed following the routine standard of care.

In the study, the ibuprofen group received an initial dose of 10 mg/kg, followed by 5 mg/kg IV daily for two doses (10-5-5 regimen). The paracetamol group received a loading dose of 20 mg/kg IV, followed by 10 mg/kg every 6 hours for 12 doses (7). The medical team conducted daily laboratory monitoring, including full blood counts, serum electrolyte levels, and liver function tests, to detect any adverse effects. Renal impairment was defined as raised creatinine (>100 micromol/L) or oliguria (<0.5 mL/kg/hour). Daily ALT and AST were deemed the best early markers to determine hepatocellular injury for the purposes of the trial.

The primary outcome was the closure or reduction of hsPDA to non-hsPDA. The secondary outcomes were to evaluate the incidence of complications of prematurity, including intraventricular haemorrhage (IVH), necrotising enterocolitis (NEC), retinopathy of prematurity (ROP), and bronchopulmonary dysplasia (BPD), by 36 weeks corrected gestational age (CGA), and to review the adverse effects of the medications. BPD severity was assessed using Ryan's classification (2006) (8). Definitive cases of NEC were identified using Bell stage >IIa criteria (Walsh and Kliegman, 1986) (9). Intraventricular haemorrhages (IVH) were classified according to the classification by Papile et al. (10). Retinopathy of prematurity (ROP), severity was interpreted in line with the International Committee for the Classification of ROP (2005) (11). As part of the pilot design, recruitment, retention and data completeness were evaluated.

Following commencing trial medication, infants in the PAIR trial received standard care and monitoring for persistent PDA. One course of open-label treatment with ibuprofen or paracetamol was allowed for those not responding to trial medications. While concerns existed about contaminating the trial, the approach reflected real-world practice and was designed to ensure that infants received necessary treatment without affecting the primary outcome results. The trial followed CONSORT Extension for Pilot and Feasibility Trials.

### Statistical analysis

All analyses were conducted on an intention-to-treat basis, including all randomised patients, with sensitivity analyses for missing data or protocol violations on the primary outcome. Baseline characteristics were presented with appropriate descriptive statistics and exploratory comparative tests, the Mann–Whitney U test for non-parametric continuous data and Fisher's exact test for discrete data. The primary and secondary outcomes were presented as proportions, along with 95% binomial confidence intervals for each group, and Fisher's exact test was used for comparisons. An exploratory univariate logistic regression analysis was used to investigate the effects of physiological and haemodynamic variables on the primary outcome, with infant characteristics as predictors. The secondary outcome analysis at 36 weeks included a sensitivity analysis for early deaths. Early participant deaths were defined as deaths occurring before 36 weeks of corrected gestational age. Analyses were conducted using R (v4.2.1), with a significance threshold of *p* < 0.05.

## Results

**Figure 2:**
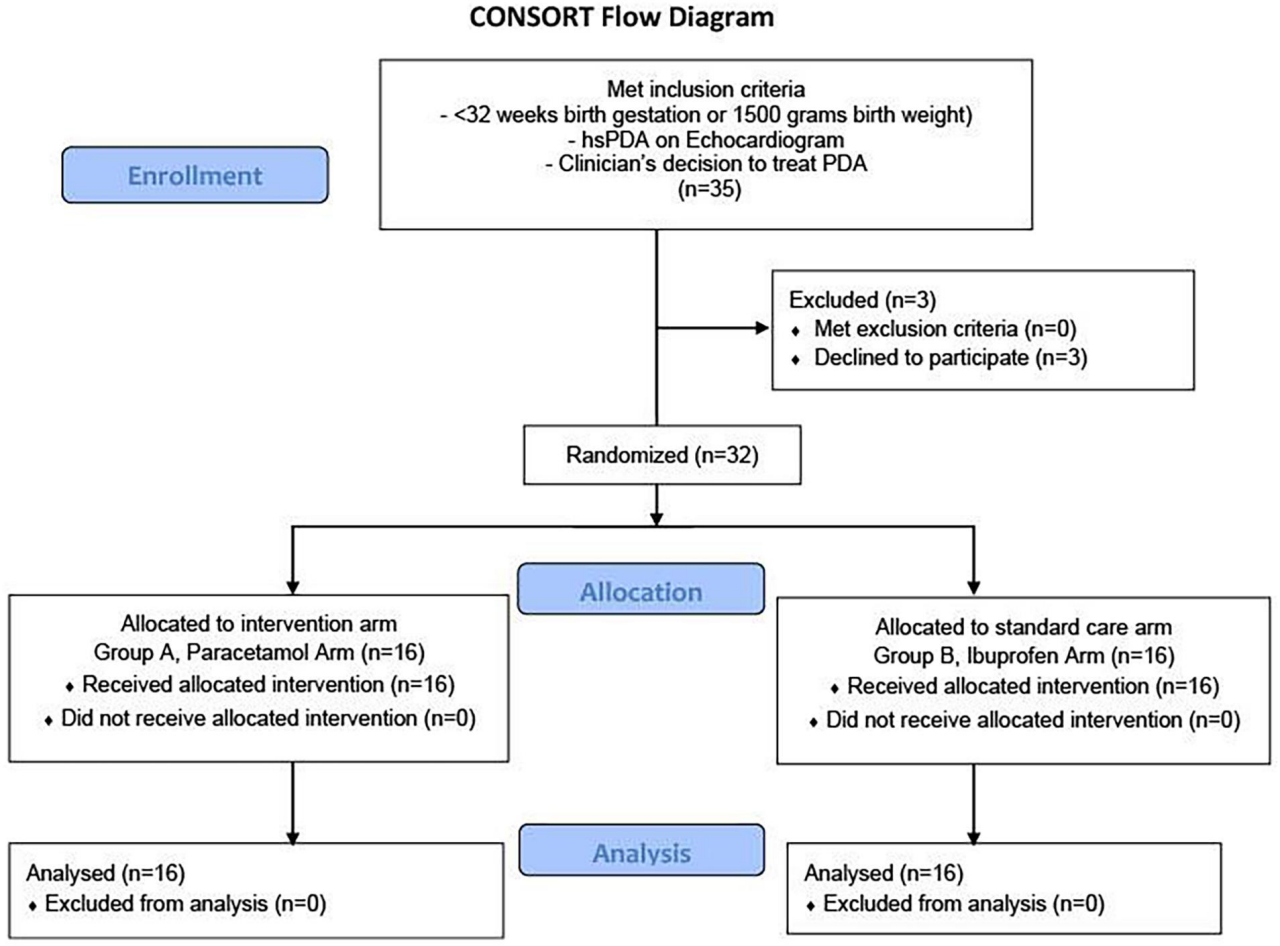
CONSORT diagram for trial recruitment.

Over a two-year period, 32 infants were enrolled for PDA treatment. Figure 2 shows the CONSORT diagram for trial recruitment. Four hundred and seventy infants with a gestation of less than 32 weeks were admitted to the NICU. Thirty-five infants (7.4%) met the inclusion criteria. The research team approached all thirty-five infants and introduced them to the study. Three families declined consent, while parents of thirty-two infants consented and successfully enrolled in the trial (91.4%). All infants who participated in the trial completed the study, resulting in a 100% retention rate, with the only data loss resulting from deaths. The study began on September 2, 2021, and concluded with the last recruit on August 16, 2023. No serious protocol breaches or significant amendments were made during the study. However, three minor amendments were implemented, and the relevant authorities, including the REC and MHRA, were notified. One participant received an extra dose of paracetamol, leading to a minor protocol deviation. A minor amendment was made to clarify the semantics regarding IV paracetamol dosing to prevent future misunderstandings in the trial.

The median [interquartile ranges (IQR)] for gestational age, birth weight, and age of treatment of the babies enrolled in the trial were 26.5 (25.2–27.4) weeks, 846 (682–1,041) g, and 10.5 (6.0–14.0) days, respectively. Baseline infant and maternal characteristics were similar across both groups, as shown in Table 1, except for pre-randomisation ventilated infants, of which a larger proportion of babies in the ibuprofen arm were ventilated (93.8% vs. 56.3%, *p* = 0.037). However, a trend toward smaller, more premature babies, requiring more oxygen and ventilation was observed in the ibuprofen group (Table 1). The effect of infant characteristics on the primary outcome was explored using univariate logistic regression analysis for hypothesis generating purposes. No statistically significant relationships were indicated and the 95% confidence intervals around the odds ratios tended to be wide, securely encompassing one.

**Table 1:** Maternal and infant characteristics.

Maternal and infant characteristics	Ibuprofen-group (*n* = 16)	Paracetamol-group (*n* = 16)	*p*-value
Maternal age in years^b^	32.0 (26.3–35.5)	30.5 (24.0–34.3)	0.792
White ethnicity^c^	11 (68.8%)	12 (75.0%)	1.000
Maternal pregnancy-induced hypertension^c^	2 (12.5%)	2 (12.5%)	1.000
Maternal diabetes^c^	0 (0.0%)	0 (0.0%)	1.000
Cox-inhibitors antenatally^c^	3 (18.8%)	3 (18.8%)	1.000
Magnesium sulphate antenatally^c^	16 (100.0%)	13 (81.3%)	0.226
Significant maternal illness^c^^,^^d^	4 (25.0%)	4 (25.0%)	1.000
Two doses of Antenatal steroids^c^	11 (68.8%)	5 (31.3%)	0.076
Maternal serology^c^	8 (50.0%)	8 (50.0%)	1.000
Normal antenatal scans^c^	16 (100.0%)	16 (100.0%)	1.000
Infant gestational age in weeks^b^	25.7 (24.4–27.1)	27.0 (26.4–27.4)	0.220
Infant gestation ≤28 weeks^c^	13 (81.3%)	16 (100.0%)	0.226
Birth weight in grams^b^	763.0 (544.5–925.2)	1,010.5 (789.2–1,047.0)	0.147
Birth weight z score^b^	−0.35 (−1.195 to 0.273)	0.60 (−0.245 to 0.743)	0.147
Birth weight ≤1,000 g^c^	13 (81.3%)	7 (43.8%)	0.066
Male sex^c^	11 (68.8%)	11 (68.8%)	1.000
Surfactant at birth^c^	16 (100.0%)	15 (93.8%)	1.000
Apgar score at 5 mins^b^	6 (5–7)	7 (5–7)	0.946
Worst base deficit within 1 h after birth^b^	−6.10 (−8.80 to −4.20)	−5.40 (−8.30 to −5.20)	0.878
Mechanically ventilated pre-randomisation^c^	15 (93.8%)	9 (56.3%)	0.037^a^
Oxygen requirement pre-randomisation^b^	37.50 (24.00–47.50)	26.50 (24.75–31.00)	0.062

a *p* < 0.05 statistically significant.

b Median (IQR), Mann–Whitney *U* test.

c Count (%), Fisher's exact test.

d Maternal illness requiring HDU/ITU admission.

Figure 2 describes the CONSORT diagram for trial recruitment. Table 1 depicts baseline infant and maternal characteristics. Table 2 shows the primary outcome, highlighting the number of babies in each group who responded to PDA treatment. Among the infants who had a reduction to non-hsPDA, PDA closed completely in 2 infants (12.5%) in the ibuprofen group and 4 infants (25.0%) in the paracetamol group (*p* = 0.654).

**Table 2:** Primary outcome by treatment group.

hsPDA to non-hsPDA	Ibuprofen-group *n* = 16	Paracetamol-group *n* = 16	Total	*p*-value^a^
Total Count, percentage	4 (25.0%)	6 (37.5%)	10 (31.3%)	0.704
95% binomial confidence intervals	(7.3%–52.4%)	(15.2%–64.6%)	(16.1%–50%)	

a Fisher's exact test.

The secondary outcomes assessed the incidence of complications of prematurity in each treatment group. Table 3 shows the incidence of complications of prematurity in each treatment group. A trend toward a higher BPD-free survival rate was observed in the paracetamol group (25.0%) compared with the ibuprofen group (6.3%). No statistically significant differences were found, except for ROP requiring treatment and ROP discharged to specialised care, where no cases were observed in the paracetamol group compared with 5 in the ibuprofen group. However, the confidence intervals were wide (0.0–41.0 vs. 18.7%–81.3%), with a borderline *p*-value of 0.044, suggesting that this result should be treated with caution.

**Table 3:** Incidence of complications of prematurity in each treatment group at 36 weeks CGA.

Complications	Ibuprofen-group (*n* = 16) count (%)	Paracetamol-group (*n* = 16) count (%)	*p*-value^d^
BPD^a^, overall	14 (87.5%)	11 (68.8%)	0.330
(i) BPD-free survival	1 (6.3%)	4 (25.0%)	0.330
(ii) Severe BPD^b^	13 (92.9%)	8 (72.7%)	0.288
(iii) Babies discharged on home O2	8 (57.1%)	6 (54.5%)	1.000
IVH^a^	4 (25.0%)	1 (6.3%)	0.333
(i) Complications from IVH^c^ at discharge	4 (100.0%)	1 (6.3%)	0.333
(ii) Needing specialist follow-up at discharge	0 (0.0%)	1 (100.0%)	1.000
NEC^a^ (Bell's stage ≥2a)	4 (25.0%)	2 (12.5%)	0.654
(i) NEC requiring surgery	1 (25.0%)	1 (50.0%)	1.000
(ii) Needing specialist follow-up at discharge	0 (0.0%)	0 (0.0%)	1.000
ROP^a^	10 (62.5%)	7 (43.8%)	0.462
(i) ROP requiring treatment	5 (50.0%)	0 (0.0%)	0.044
(ii) Discharged to specialist follow-up	5 (50.0%)	0 (0.0%)	0.044

a BPD, bronchopulmonary dysplasia; IVH, intraventricular haemorrhage; NEC, necrotising enterocolitis; ROP, retinopathy of prematurity.

b Severe BPD—BPD requiring FiO2 > 30%, non invasive or invasive respiratory support.

c Complication from IVH—periventricular hemorrhagic infarction or hydrocephalus.

d Fisher's exact test.

The adverse effects of trial medications were reviewed in three main domains: renal, hepatic, and bleeding (both gastrointestinal and non-gastrointestinal). In each group, one baby experienced pulmonary haemorrhage. One infant in the ibuprofen group experienced transient renal impairment, which resolved spontaneously within three days after completing the ibuprofen treatment. Serial laboratory parameters remained normal after trial medications. All participants were monitored for gastrointestinal bleeding, and no incidents occurred.

Table 4 summarises the PDA status during hospitalisation for all infants in the PAIR trial who had hsPDA from admission to discharge. A total of 11 infants, six in the ibuprofen group and five in the paracetamol group, received open-label treatment for persisting hsPDA. This treatment included a second three-day course of either ibuprofen or paracetamol. Two infants from each group underwent PDA closure following this treatment, whilst a further one each required invasive procedures. At the time of discharge, 43.8% of infants from the total ibuprofen group and 50% in the paracetamol were found to have a small or insignificant PDA. These were subsequently followed up and monitored by a paediatric cardiologist in an outpatient clinic.

**Table 4:** Outcomes of various treatment options during routine clinical care.

Treatment	Ibuprofen group count (%)	Paracetamol group count (%)	*p*-value^a^
PDA closed following the randomised treatment	2 (12.5%)	4 (25%)	0.655
Open-label treatments	6 (37.5%)	5 (31.3%)	1.000
PDA closed following open-label treatment	2 (33.3%)	2 (40%)	1.000
Surgical ligation	1 (6.3%)	0 (0.0%)	1.000
Medical device closure	0 (0.0%)	1 (6.3%)	1.000
Total PDA at discharge (small)	7 (43.8%)	8 (50%)	1.000

a Fisher's exact test.

## Discussion

The prophylactic treatment of PDA within 12–24 h and early, selective, targeted treatment within 72 h have failed to provide favourable outcomes. Current clinical practice is therefore increasingly leaning towards conservative, expectant management. It appears more rational to provide targeted interventions to those infants who are symptomatic or at very high risk based on specific clinical and echocardiographic criteria. The PAIR trial examined rescue treatment for premature infants with established hsPDA beyond 72 h of life, continuing through the first 4 weeks of life.

An observational and expectant management approach was implemented for all eligible participants with PDA. Out of 470 infants assessed for eligibility, only 35 (7.4%) met the inclusion criteria for rescue treatment and participated in the trial. Notably, 91.4% of the parents approached agreed to participate, reflecting a strong willingness to enrol in the study. Although one minor protocol deviations were reported to the MHRA, there were no instances of missing data or loss to follow-up during the trial.

Paracetamol administration reduced hsPDA to non-hsPDA in 37.5% of cases, whereas ibuprofen resulted in a 25.0% conversion rate. In the paracetamol group, 25.0% of hsPDAs closed, compared to 12.5% in the ibuprofen group. No statistically significant difference in effectiveness was found between the two treatments.

At the onset of the PAIR trial, only two RCTs, conducted by El Mashad et al. and Tauber et al., assessed the effectiveness of intravenous (IV) paracetamol vs. IV ibuprofen for the rescue treatment of PDA (1, 12, 13). In the study by El Mashad et al., 100 infants (born at less than 28 weeks’ gestation or weighing less than 1,500 grams at birth) were treated with paracetamol at a dose of 15 mg/kg every 6 h for three days. In the ibuprofen group, a similar number of infants received the conventional 10-5-5 regimen. The treatment window was the first two weeks of life. The reported closure rates of PDA following the first treatment were 80% with paracetamol and 77% with ibuprofen, while our study showed significantly lower closure rates. Both Tauber et al. and El Mashad et al. administered a higher cumulative dose of paracetamol (180 mg/kg) over three days during the first two weeks of life. In contrast, the PAIR trial used a lower cumulative dose of paracetamol (130 mg/kg) over three days, with a treatment window of the first four weeks of life. The ibuprofen doses in these RCTs were similar to those used in the PAIR trial (10-5-5 regimen). Furthermore, the El Mashad study reported considerable side effects associated with ibuprofen, which were not observed in the PAIR trial. Even if higher doses of paracetamol were deemed more effective, they do not explain the differing ibuprofen closure rates since both trials used similar dosages. The variations might be attributed to the timing of medication administration and the specific populations of infants studied. The median birth weight in the El Mashad study was approximately 1.1 kg, which is above the third quartile for our study (median birth weight: 846.5 g, IQR: 686.2–1,041.5), indicating that, overall, our babies were lighter. Additionally, the median age at treatment initiation in the El Mashad study was about 2.7 days. This was much earlier than the PAIR trial, where the median age of infants receiving trial medications was 10.5 days (12). The success of the El Mashad study can also be linked to a general observation in multiple studies that earlier treatment yields higher success rates.

The second RCT by Tauber et al. found that paracetamol was more effective than ibuprofen for closing PDA in the first two weeks of life, reporting that ibuprofen was ineffective (0%) compared to paracetamol (40%) (13). However, the small sample size of 5 infants in each arm makes it difficult to draw meaningful conclusions

The Baby-OSCAR trial (2021) found that only 55.5% of infants born between 23 and 28+ 6 weeks of gestation who received ibuprofen within 72 h of birth had a closed ductus arteriosus or a small, insignificant PDA at three weeks of age (14). In contrast, treatment initiation in the PAIR trial occurred relatively late, with a median of 10.5 days. Research indicates that the effectiveness of NSAIDs like ibuprofen on ductal constriction is greatest when administered within the first day after birth and diminishes with increasing postnatal age (15–17). Delaying intervention for an established PDA often leads to less effective treatment. This helps explain the lower efficacy rates observed in the PAIR trial, where ibuprofen had a success rate of only 25.0% and paracetamol 37.5%, as these treatments were used in a later rescue setting. The study's findings highlight the real-world effectiveness of interventions for symptomatic hsPDA during the first four weeks of life.

In evaluating the secondary outcomes of the PAIR trial, it is important to discuss the observed trends. The rate of BPD-free survivors was higher in the paracetamol group (25.0%) compared to the ibuprofen group (6.3%). Severe BPD occurred more frequently in the ibuprofen group (81.3% vs. 50.0%), and a greater proportion of infants in this group were discharged on home oxygen (50.0% vs. 37.5%). While this data may suggest a possible link between ibuprofen use and an increased incidence of BPD, caution is warranted in interpreting these results due to the small sample size, which limits the ability to draw meaningful conclusions.

Despite randomisation, infants in the ibuprofen group tended to be more premature, with a median gestational age of 25.7 weeks (IQR 24.4–27.1) compared to 27.0 weeks (IQR 26.4–27.4) in the paracetamol group. However, the upper quartiles for gestational age were very similar between the two groups. The babies in the ibuprofen group also tended to be smaller, with a median weight of 763 g (IQR 544.5–925.2) compared to 1,010 g (IQR 789.2–1,047.0). Moreover, a larger percentage of these infants were on ventilation (94% vs. 54%) and required more oxygen (37% vs. 26%). These factors could have independently or collectively influenced the risks of respiratory morbidity and the incidence of BPD. The absence of statistical significance may be attributed to the small sample sizes; however, the observed differences are clinically relevant. It is noteworthy that a recent European trial, the Beneductus trial, which examined the use of ibuprofen within 72 h of birth for treating PDA, also indicated a higher incidence of BPD among the ibuprofen-treated group. Furthermore, a recent systematic meta-analysis on early treatment with ibuprofen (within 72 h) concluded that there was a higher incidence of all-cause mortality associated with ibuprofen use compared to placebo or expectant management (18). The American Academy of Pediatrics published new guidelines for PDA management in April 2025. These findings underscore the need for a reevaluation of the use of ibuprofen in medical management, especially since one of its intended purposes is to prevent BPD. Additionally, in the PAIR trial, although no significant difference in ROP (retinopathy of prematurity) occurrence was observed between the two groups, the ibuprofen group had a higher ROP severity requiring treatment and specialist follow-up (50% vs. 0%; statistically significant). Variations in ROP severity between the two groups may have been influenced by the greater number of premature and ill infants in the ibuprofen group. This warrants further investigation in a larger study.

One infant in each treatment group died from severe NEC before reaching 32-week CGA. Both babies were very ill (ventilated and on several inotropes), and no temporal relationship between the use of trial medications and the incidence of NEC was established.

One important secondary objective of this study was to examine potential adverse effects associated with the treatment. The results revealed no significant differences in adverse effects, including predictable and unpredictable types. Serial laboratory parameters remained within the normal reference ranges following administration of the trial medications. All participants were assessed for gastrointestinal bleeding, and no incidents occurred. While paracetamol is generally not linked to gastrointestinal side effects, Shahmirzadi et al. (5) reported an increased incidence and severity of gastrointestinal bleeding, feed intolerance, and NEC in their RCT involving 23 infants who were administered paracetamol (19). The authors expressed legitimate concerns regarding the safety profile of paracetamol. This finding highlights the need for further research to address these concerns. Katasaras et al. (1) reviewed 12 randomised controlled trials (RCTs) on intravenous (IV) paracetamol for the treatment of PDA. Except for the Shahmirzadi et al. study, the other eleven RCTs, which included 394 infants, reported no serious adverse effects.

One course of open-label medical treatment was permitted for infants who continued to show signs of hsPDA despite receiving the trial medications. In total, 11 of 32 infants received this open-label treatment. The treatment included a second three-day course of either ibuprofen or paracetamol. At the time of discharge, 43.8% of infants in the ibuprofen group and 50% in the paracetamol group were found to have a small or insignificant PDA. They were subsequently followed up and monitored by a paediatric cardiologist in an outpatient clinic.

### Strengths of the trial

The PAIR trial remains relevant in a clinical context. The study utilised a robust methodology with clearly defined primary and secondary outcomes. The study groups represented the at-risk clinical population, reflecting real-world challenges and clinical scenarios. To this end, PAIR is the first study to compare the efficacy and safety of paracetamol against ibuprofen in the first four weeks of life. The trial achieved its primary aim of assessing feasibility. The trial successfully met the recruitment target within the time frame and retained all recruited babies. Data were complete at the end, with minor deviations, which will be used to provide guidance in future trial conduct, a learning that will be valuable in the larger study.

### Limitations of the trial

The sample size of 32 infants is small and was not designed to provide definitive conclusions about the efficacy and safety of paracetamol. However, the pilot PAIR trial provided valuable insights for the full study design, demonstrating it is both feasible and desirable.

Due to funding limitations, double-blinded allocation concealment could not be implemented. To minimise bias, echocardiograms were reviewed and reported by three designated specialist paediatric cardiologists. Although they were all trained to apply the trial criteria consistently, interobserver variability inherent to the technique cannot be completely ruled out. We concur that a larger trial should also include measures to evaluate inter-rater reliability.

In the trial, 11 babies (34.4%) received additional open-label treatment due to persistent symptoms of hsPDA after the initial three days of study medication. While this approach mirrors real-world clinical practice, it has the potential to influence secondary outcomes; therefore, careful consideration should be given to the interpretation of these results.

## Conclusions

This single-centre, prospective pilot RCT did not demonstrate any statistically significant differences in the effectiveness or safety of IV paracetamol compared to IV ibuprofen for the treatment of hsPDA in preterm infants. No significant adverse effects were noted in either arm. Ninety-one per cent of eligible neonates were recruited and enrolled following parental consent, demonstrating the trial's feasibility. This pilot trial was the first step in achieving a baseline comparative analysis, ascertaining the level of adverse effects in this vulnerable population and demonstrating that paracetamol may be safe to use. The trial supports the design and execution of a multicentre study.

## Data Availability

The original contributions presented in the study are included in the article/Supplementary Material, further inquiries can be directed to the corresponding author.

## References

[1] Katsaras DN Katsaras GN Chatziravdeli VI Papavasileiou GN Touloupaki M Mitsiakos G Comparative safety and efficacy of paracetamol versus non-steroidal anti-inflammatory agents in neonates with patent ductus arteriosus: a systematic review and meta-analysis of randomized controlled trials. Br J Clin Pharmacol. (2022) 88(7):3078–100. 10.1111/bcp.15291 35203104

[2] Ambalavanan N Aucott SW Salavitabar A Levy VY. Committee on fetus and newborn, section on cardiology and cardiac surgery; patent ductus arteriosus in preterm infants. Pediatrics. (2025) 155(5):e2025071425. 10.1542/peds.2025-071425 40288780

[3] Noureldein M Hu K Groucutt J Heaver R Gurusamy K. Paracetamol for patent ductus arteriosus in preterm infants: a UK national survey. J Matern Fetal Neonatal Med. (2022) 35(7):1408–11. 10.1080/14767058.2020.1752652 32290734

[4] Mukherjee A Jadhav V Gupta A. Off-label use of paracetamol in managing patent ductus arteriosus across neonatal intensive care units in the UK. Arch Dis Child Fetal Neonatal Ed. (2020) 106:113–4. 10.1136/archdischild-2020-320207 33106275

[5] Shahmirzadi G Nooripour S Ziari A Danaei N. Comparison of gastrointestinal complications of paracetamol and ibuprofen in the management of infants with patent ductus arteriosus: a randomized clinical trial study. Int J Prev Med. (2021) 12(1):48. 10.4103/ijpvm.IJPVM_387_19 34211679 PMC8223912

[6] Van Laere D Van Overmeire B Gupta S El Khuffash A Savoia M McNamara PJ Application of NPE in the assessment of a patent ductus arteriosus. Pediatr Res. (2018) 84(Suppl 1):46. 10.1038/s41390-018-0077-x 30072803 PMC6257219

[7] Allegaert K Palmer GM Anderson BJ. The pharmacokinetics of intravenous paracetamol in neonates: size matters most. Arch Dis Child. (2011) 96(6):575–80. 10.1136/adc.2010.204552 21317433

[8] Ryan RM. A new look at bronchopulmonary dysplasia classification. J Perinatol. (2006) 26(4):207–9. 10.1038/sj.jp.7211449 16570079

[9] Walsh MC Kliegman RM. Necrotizing enterocolitis: treatment based on staging criteria. Pediatr Clin N Am. (1986) 33(1):179–201. 10.1016/S0031-3955(16)34975-6 PMC7131118 3081865

[10] Papile L-A Burstein J Burstein R Koffler H. Incidence and evolution of subependymal and intraventricular hemorrhage: a study of infants with birth weights less than 1,500 gm. J Pediatr. (1978) 92(4):529–34. 10.1016/S0022-3476(78)80282-0 305471

[11] El-Mashad AE-R El-Mahdy H El Amrousy D Elgendy M. Comparative study of the efficacy and safety of paracetamol, ibuprofen, and indomethacin in closure of patent ductus arteriosus in preterm neonates. Eur J Pediatr. (2017) 176:233–40. 10.1007/s00431-016-2830-7 28004188

[12] Tauber KA King R Colon M. Intravenous acetaminophen vs intravenous ibuprofen to close a patent ductus arteriosus closure: a pilot randomized controlled trial. Health Sci Rep. (2020) 3(3):e183. 10.1002/hsr2.183 32775700 PMC7405412

[13] Sellmer A Bjerre JV Schmidt MR McNamara PJ Hjortdal VE Høst B Morbidity and mortality in preterm neonates with patent ductus arteriosus on day 3. Arch Dis Child Fetal Neonatal Ed. (2013) 98(6):F505–F10. 10.1136/archdischild-2013-303816 23893268

[14] Hundscheid T Onland W Kooi EM Vijlbrief DC de Vries WB Dijkman KP Expectant management or early ibuprofen for patent ductus arteriosus. N Engl J Med. (2023) 388(11):980–90. 10.1056/NEJMoa2207418 36477458

[15] Clyman RI Hills NK Liebowitz M Johng S. Relationship between duration of infant exposure to a moderate-to- large patent ductus arteriosus shunt and the risk of developing bronchopulmonary dysplasia or death before 36 weeks. Am J Perinatol. (2020) 37(02):216–23. 10.1055/s-0039-1697672 31600791 PMC9940607

[16] Clyman RI. Ibuprofen and patent ductus arteriosus. N Engl J Med. (2000) 343(10):728–30. 10.1056/NEJM200009073431009 10974138

[17] Gupta S Subhedar NV Bell JL Field D Bowler U Hutchison E Trial of selective early treatment of patent ductus arteriosus with ibuprofen. N Engl J Med. (2024) 390(4):314–25. 10.1056/NEJMoa2305582 38265644 PMC7615774

[18] Erzinger G Rajith G Torres MH Gauza M Mansuri Z Cardoso SM. Early drug treatment in preterm patients with large patent ductus arteriosus at 28 weeks or less gestational age: systematic review and meta-analysis. J Perinatol. (2024) 45:1–5. 10.1038/s41372-024-02154-4 39420074

[19] Prematurity ICftCoRo. The international classification of retinopathy of prematurity revisited. Arch Ophthalmol. (2005) 123(7):991–9. 10.1001/archopht.123.7.991 16009843

