# Supplementary material for: Paracetamol or ibuprofen? A pilot study comparing rescue therapy for PDA in preterm infants within the first month
Source: Front Pediatr. 2026 Jan 2;13:1717284. doi: 10.3389/fped.2025.1717284 (PMC12808359; doi:10.3389/fped.2025.1717284)
Supplement: Supplementary file 1 [file Datasheet1.pdf]

### (A) Rationale for the clinical trial

In the UK, intravenous paracetamol is approved for use in infants 32 weeks of gestation or older to manage pain or fever. Although there is consideration for using paracetamol as a medical treatment for hsPDA as an alternative to traditional NSAID treatments, it is not currently licensed for this purpose in the UK. RCTs have investigated the effectiveness and safety of paracetamol in premature neonates. However, establishing best practices has been challenging due to significant variability in how these trials have been conducted. Consequently, there remains significant variation in clinical practice regarding using paracetamol for treating PDA in the UK.[1,2]

Before the trial began, we conducted a national survey across 63 level 3 neonatal intensive care units (NICUs) in the UK between February and April 2020.[2] The response rate was 95% (60 out of 63). The survey revealed that 82% of NICUs currently use paracetamol as an off-label medication to manage hsPDA. 65% of the NICUs used paracetamol as a second-line treatment when ibuprofen was ineffective, while 10% used paracetamol as a first-line medication. Furthermore, 73% of the NICUs reported using only the intravenous form of paracetamol. The survey also found that 51% of the NICUs administered paracetamol at a dosage of 15 mg/kg/dose every 6 hours for three days, with 20% using the same dosage for five days and 2% using 20 mg/kg/dose every 6 hours for 3 days. Some NICUs also had uncertainty about the appropriate dosage of paracetamol.[226] The survey results showed a lot of variation in the doses, treatment duration, and monitoring of infants receiving paracetamol in different NICUs in the UK. Opinions on paracetamol's effectiveness and side effects varied, with 47% of respondents expressing uncertainty about its success rate. These findings underscore the importance of establishing strong evidence to support consistent guidelines for managing hsPDA. The survey also demonstrated substantial interest among NICUs in the UK to participate in a randomised controlled trial (RCT) comparing the effectiveness and safety of intravenous paracetamol with intravenous ibuprofen. [2,3]

Following the national survey, a comprehensive literature review was conducted. The literature review was specifically tailored to focus on RCTs, upholding rigorous standards and ensuring the highest quality of evidence. The variability in dosage regimens and monitoring of infants on IV paracetamol was analysed using the findings from the RCTs. Safety concerns associated with paracetamol treatment were identified through additional searches of case reports, case series, cohort studies, and observational studies to ensure that nothing was missed. However, it is crucial to acknowledge that many of these RCTs have shortcomings, including inconsistencies in the definition of hsPDA, variations in the parameters used to assess hsPDA through echocardiograms, differing criteria for participant inclusion in the studies, small sample sizes, and the absence of intention-to-treat analyses.

Four databases that met the selection criterion in Table 4 were searched for evidence from the literature. OVID EMBASE (1974 to 2021 February 28) and OVID MEDLINE (1946 to 2021 February 28) databases were searched to identify relevant studies. Additionally, searches were conducted in the Cochrane Database and the ClinicalTrials.gov database. A direct head-to-head comparison was made between intravenous (IV) paracetamol and IV ibuprofen for managing hsDPA. Special attention was given to examining all RCTs that used intravenous paracetamol to uncover any safety issues. An additional search was also made in the PubMed database. No additional results were obtained.

The literature search utilised the terms "ibuprofen" and "ibuprofen. ti, ab," "patent ductus arteriosus," "PDA," "acetaminophen," "paracetamol," "IV," and "intravenous administration." Two investigators ran the searches to minimise errors and cross-checked and validated the search criteria and results.

Table 1 lists the inclusion and exclusion criteria deployed in the literature search. In total, 76 papers were identified from the databases mentioned. First, the titles were screened, followed by the abstracts to filter out irrelevant papers. Thirty-five papers were read, and 29 were excluded for reasons cited in Figure 1. Further, six papers were read in full, and only four were chosen for this review. The PRISMA flowchart (Figure 1) outlines the process and rationale behind the selection of these studies.

**Table 1*: Inclusion and Exclusion Criteria for Database Search***

| Parameters | Inclusion Criteria | Exclusion Criteria |
|---|---|---|
| **Population** | Preterm infants diagnosed with patent ductus arteriosus | Other congenital heart conditions |
| **Intervention** | *IV paracetamol | Oral paracetamol<br>[$]Vs. Oral ibuprofen<br>Vs. IV indomethacin<br>Vs Oral indomethacin |
| **Control/Comparator** | IV Ibuprofen | Oral ibuprofen |
| **Outcome** | Closure of patent ductus arteriosus, Safety, efficacy, liver impairment/failure, renal impairment/failure, gastrointestinal perforation, gastrointestinal bleeding, **IVH, BPD, ROP, NEC | None |
| **Study Design** | Randomised controlled trials with full-text articles<br>English Language | Systematic reviews and meta-analyses.<br>Observational studies, case-control studies, Case series, case reports, and cohort Studies.<br>Expert opinion, non-published papers, full-text unavailable, other languages |

**IV Intravenous, [$]Vs. versus, **IVH Intraventricular haemorrhage, BPD Bronchopulmonary dysplasia, ROP Retinopathy of prematurity, NEC Necrotising enterocolitis*

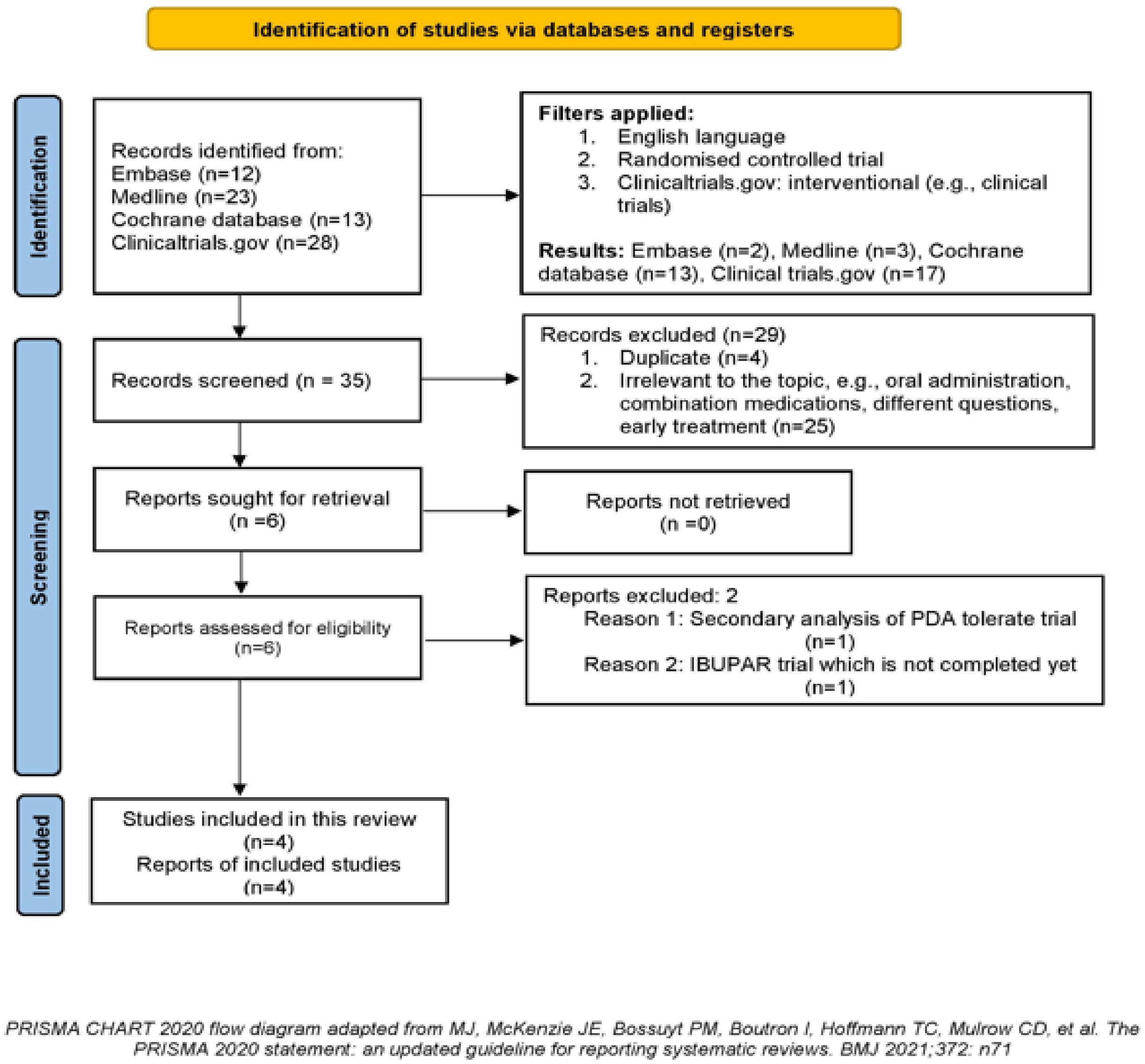


**Figure 1: PRISMA Chart for Literature Search and Identifying Databases and Registers**

Table 2 lists all the studies in which IV paracetamol has been used and compared with placebo or other NSAIDs for PDA treatment. The four trials that compared IV paracetamol and IV ibuprofen head-to-head are El-Mashad et al., Tauber et al., Jafari et al., and Dani et al.[4,5,6,7]

El-Mashad et al.[4] (Egypt) compared the efficacy of IV paracetamol, IV ibuprofen and IV indomethacin in the first two weeks of life for treating hsPDA diagnosed by echocardiography and clinical examination. 100 premature infants with gestational age less than 28 weeks or birth weight less than 1500 grams were recruited in each arm. Echocardiographic criteria of hs-PDA were left atrial dilatation (left atrial: aortic root >1.6), diastolic turbulence (backflow) on Doppler in the pulmonary artery, internal diameter of duct >1.5 mm, and reverse end diastolic flow in the descending aorta/mesenteric artery. These indices were taken from the work of McNamara et al. in 2007.[214] An IV dose of 15 mg/kg of paracetamol every 6 hours for three days was administered. For ibuprofen, an initial IV infusion of 10 mg/kg was followed by a daily dose of 5 mg/kg for two days (10-5-5). After the first treatment course, the closure rate was 80% with paracetamol therapy and 77% with ibuprofen. The researchers also observed a significant decrease in platelet levels, a significant increase in creatinine and BUN (Blood urea nitrogen), and significant hyperbilirubinemia, as well as some degree of gastrointestinal bleeding, with ibuprofen. The renal effects (increased creatinine, BUN, and decreased urine output) were not seen in the paracetamol group. No rise in the liver enzymes ALT/AST were noted with paracetamol.

Tauber et al.[7] (USA) conducted an even smaller study with five infants in each group (ibuprofen and paracetamol). Infants between 23 weeks and 29+6 weeks of gestation at birth with a hsPDA diagnosed by echocardiogram within the first two weeks of life were included. The DA was graded as large, moderate, small, or closed, taking into consideration the LA: Ao ratio, the ductal diameter, and the Doppler flow pattern through the PDA. The IV ibuprofen group received three doses: a loading dose of 10 mg/kg/dose on the first day and a 5 mg/kg/dose on the second and third days (popularly called a 10-5-5 regimen). The IV paracetamol group received 12 doses: 15mg/kg/dose administered every 6 hours (for a total of three days). The primary outcome was PDA closure. Adverse effects such as a rise in creatinine, AST/ALT, and fractionated bilirubin levels were within normal limits in both groups before, during, and after the trial medication course. There were no cases of NEC or GI bleeding. PDA closure was observed in two of the five infants in the IV paracetamol group (40%) vs. zero in five infants (0%) in the IV ibuprofen group.

Dani et al. (Italy) used an inclusion criterion of gestational age between 25+0 and 31+6 weeks, with echocardiographic evidence of hsPDA between 24 and 72 hours of life.[6] The diagnosis of hsPDA was made by echocardiographic demonstration of a ductal left-to-right shunt, with a left atrium-to-aortic root ratio >1.3 or a ductal size >1.5 mm and excluding the cases in which the closing flow pattern suggested a restrictive PDA. These echocardiogram parameters were obtained from studies by Varvarigou et al. (1996) and Su et al. (2008).[8, 9] The dose of paracetamol used was 15mg/kg/dose, administered intravenously every six hours for three days. The dose of ibuprofen was 10mg/kg/dose loading, followed by 5mg/kg/dose for two more days (10-5-5). The study's primary outcome was the closure of hsPDA after the first course of treatment with paracetamol compared to ibuprofen. 52 infants received paracetamol treatment, and 49 infants received ibuprofen. They concluded that paracetamol was less effective in closing hsPDA than ibuprofen (52% vs. 78%; P=0.026). The occurrence of adverse events was similar in the paracetamol and ibuprofen groups.

Jafari et al.[5] (Iran) conducted a trial involving all low-birth-weight infants at the postnatal age of 48-72 hours with moderate to large PDA (demonstrated on echocardiogram). A total of 30 premature infants (with gestational age between 28 and 34 weeks) were studied. The efficacy of IV paracetamol was similar to that of IV ibuprofen in treating PDA. The safety aspects of the medications were not studied.

The findings on efficacy from El-Mashad et al., Dani et al., and Tauber et al. showed inconsistencies. The variability among these RCTs is also very apparent. El-Mashad et al. observed significant side effects in 100 infants treated with ibuprofen, while the other trials involving 68 infants did not. From a physiological standpoint, PDAs are unlikely to become haemodynamically significant in the first few days of life due to high pulmonary vascular resistance (PVR) during this period (section 3.9.6). A PDA usually develops into a hsPDA beyond the first 48-72 hours of life. Jafari et al. and Dani et al. conducted their studies in the early postnatal period within 72 hours of birth, when PDAs are unlikely to become haemodynamically significant (hsPDA).[10,11] While theoretically, El-Mashad et al. and Tauber et al. conducted their trial to evaluate the

efficacy of the treatment for hsPDA, their studies did not extend beyond the first two weeks of age. It is not uncommon to administer medical treatment for hsPDA in extremely preterm infants during their first month of life if there are clinical concerns. This approach is often taken because if the medical management of PDA is unsuccessful, more invasive procedures like surgical ligation or device closure may be necessary. Unfortunately, the differing trial designs, timings, and methodologies of these RCTs resulted in conflicting findings on the efficacy of IV paracetamol. Consequently, they failed to provide the necessary evidence to guide clinical practice.[12]

Katsaras et al. (2022) published a systematic review and meta-analysis of all randomised controlled trials that compared the safety and efficacy of paracetamol against NSAIDs in treating PDA.[12] This review concluded that there was no significant difference in efficacy between paracetamol and NSAIDs in treating PDA. In addition, paracetamol has the added advantage of fewer side effects than other NSAIDs. [12] Interestingly, this review included 20 RCTs, of which only four published RCTs compared IV ibuprofen with IV paracetamol for PDA treatment (Table 2). These are El Mashad et al. (2016), Jafari et al. (2019), Dani et al. (2020) and Tauber et al. (2020). I have already alluded to the issues with these trials in my literature review.

It is apparent from the ongoing discussion that the evidence for the efficacy and safety of intravenous paracetamol compared with intravenous ibuprofen for the treatment of PDA in the preterm population remains limited. This is primarily due to the use of fewer trials with the intravenous preparation of the medications, heterogeneity among the trials, variability in dosages and timing of medication use, and relatively small sample sizes. Good-quality data addressing its efficacy, safety, and dosage will be of significant value to support clinicians and to ensure evidence-based, safe clinical practice.

**(B) Rationale for Pilot Trial**

No clinical trials have been conducted in the UK to compare these two medications for the treatment of PDA. It is important to note that indomethacin was not replaced by ibuprofen due to a lack of efficacy but rather because of safety concerns. If paracetamol is to replace ibuprofen as the preferred medication, it must demonstrate comparable efficacy and fewer adverse effects in treating PDA. This can only be established through a robust, double-blinded, randomised, controlled multicentre study. However, given the uncertainty surrounding the existing evidence, it would be imprudent to proceed directly to a large multicentre trial.

This pilot trial is the initial step in better understanding the role of intravenous paracetamol in treating PDA. The primary focus was on testing the trial design, including recruitment, retention, and the randomisation processes. The trial chose to include infants with a gestational age of less than 32 weeks or a birth weight of less than 1500 grams, as this group encompasses the majority of newborns who require PDA treatment. By doing this, our data will ensure external validity and represent the target population accurately. Following this trial, the goal is to use the findings to inform the design and estimate the sample size for a comprehensive multicentre study. Moreover, the trial will help prioritise outcomes and foster engagement with patients and caregivers regarding this type of research. The pilot data will not only aid in recruiting hospitals for the larger trial but will also facilitate the assessment of potential adverse effects in this vulnerable population, demonstrating whether paracetamol is safe for use.

**( C ) Selection of the route of administration of the trial medications**

The closure of PDA in extremely premature infants occurs in only about one-third of cases by day 4 of life. [13, 14] Premature infants also have less cardiopulmonary reserve compared to the bigger and more mature infants born at term, which influences their ability to cope with PDA. Therefore, these premature infants often become symptomatic after the first few days of life if they have a large ductal shunt.[15] Medical treatment of PDA works most effectively during the first week of life.[15, 16] Rarely, extremely premature infants are entirely enterally fed by the end of the first week of life. Babies at this gestation are born with an immature gut due to premature birth, making them poorly tolerant of oral feeding. Consequently, they often rely on intravenous parenteral nutrition in the first few weeks of life. Feed increments are introduced slowly, so it takes time for these infants to establish full feeds. Therefore, oral medication is not the best choice for treating these extremely low birth-weight

infants. The combination of gut immaturity, poor enterohepatic circulation, and feed intolerance in these infants likely contributes to reduced efficacy, unpredictable bioavailability, and potential toxicity.

Considering the high prevalence of hsPDA in this vulnerable group and the uncertainty about the adequate absorption of oral medications, the PAIR trial investigates the efficacy of the intravenous (IV) preparations of paracetamol and ibuprofen in PDA. As mentioned earlier, several studies have examined the effects of oral preparations on these two medications; however, very few studies have compared the efficacy of their IV formulations. [12]

In a 2019 systematic review conducted by Xiao et al., oral paracetamol was found to be effective for more mature infants weighing between 1,501 and 2,500 grams at birth.[17] In their retrospective study, Semberova et al. highlighted the impact of prematurity levels and recommended that studies on hsPDA closure should focus on the smallest and youngest infants.[13] In our survey, the majority (73%) of UK NICUs reported using only the IV preparation of paracetamol. Meanwhile, a minority (26%) of NICUs in the UK used either IV or enteral preparation, depending on the infants' fed status.[2]

Since ibuprofen is only licensed for treating PDA as an intravenous preparation in the UK, the PAIR trial adopted the same dosage regimen for easier comparison.[177] This dosing schedule is part of the standard care for infants with hsPDA at St. Mary's neonatal unit and has received consensus approval within the neonatal NWODN.[19] The dosage consists of 10 mg/kg on the first day, followed by two doses of 5 mg/kg, administered 24 hours apart (10-5-5). Paracetamol is classified as an investigational medicinal product (IMP) for the PAIR trial. Currently, no oral medications are licensed in the UK for use during this gestational period to treat PDA; therefore, the PAIR trial opted to continue using the intravenous route of administration.

**(D) Dosage of trial medications (Paracetamol)**

***1. IMP (paracetamol)***

***a) Pharmacokinetics and pharmacodynamics of paracetamol***

The half-life of paracetamol in adults is suggested to be 5.6 hours. However, most pharmacokinetic studies performed in preterm and term newborn infants found these to be 4.6 hours and 2.9 hours, respectively.[20,21]

In adults, paracetamol metabolism occurs in the liver, where the following pathways lead to the formation of non-toxic metabolites that are eventually eliminated by the kidneys.[20,21]

- **i)** Glucuronidation (45-55%)
- **ii)** Sulphation (sulphate conjugation) (20- 30%)
- **iii)** N-hydroxylation, rearrangement, followed by glutathione conjugation (<15%)

In extremely preterm infants, the primary mode of metabolism is sulphation, and the metabolism of paracetamol by glucuronidation increases with advancing gestational age.[21] As the extent of glucuronidation and sulfation depends on the gestation and maturity of infants, a uniform standard serum level across all gestations becomes hypothetical. In addition, appropriate clinical trial designs and assays incorporating robust evaluation of gestation-specific serial serum concentrations to determine the optimal dose-response threshold and toxicity have yet to be established in extremely preterm infants.[22,23]

Vasoconstriction is believed to be the main cause of renal impairment, cerebral ischemia, and NEC associated with NSAIDs. Interestingly, paracetamol has a vasodilatory effect, unlike NSAIDs, which exert a vasoconstrictive mechanism of action. This observation may clarify the reduced side effects observed with the regular use of paracetamol. While vasodilation raises concerns of clinical hypotension following paracetamol administration, this has not been a significant issue in the neonatal population.[24] It is postulated that the metabolite responsible for the vasodilatory effects of paracetamol is NAPQI (N- acetyl-p- benzoquinone imine). As less than 15% of paracetamol is metabolised via this route and the fact that NAPQI is detoxified rapidly by glutathione in the liver and excreted in the urine, its impact under physiological conditions in vivo is minimal and short-acting. [23,24,25]

***<u>2 Selection of the dose/duration of paracetamol for hsPDA treatment</u>***

Two national surveys in the UK on the use of paracetamol to treat PDA have shown a lack of uniformity in dosages used and highlighted the limitations of the current level of evidence in guiding clinical practice.[1,2]

All available RCTs on IV paracetamol were evaluated before the PAIR trial was set up, and the findings are compiled in Table 2 (before the Bibliography). A total of twelve RCTs have been conducted to investigate the efficacy of IV paracetamol in treating PDA in preterm infants. In these trials, the researchers utilised different dosing regimens. El-Mashad et al., Jafari et al., Dani et al., Davidson et al., Tauber et al., and Meena et al. administered doses of 15 mg/kg every 6 hours for three days, resulting in a cumulative dose of 180 mg/kg. On the other hand, Harkin et al. and Hochwald et al. used a loading dose of 20 mg/kg followed by 7.5 mg/kg every 6 hours for four days and 10 mg/kg every 6 hours for three days, respectively, yielding cumulative doses of 130-132.5 mg/kg over the course duration. Schindler et al. adopted a loading dose of 15 mg/kg followed by 7.5 mg/kg every 6 hours for five days, resulting in a cumulative dose of 157.5 mg/kg. Shahmirzadi et al. and Ghaderian et al. administered 15 mg/kg/dose every 6 hours for two days, resulting in a cumulative dose of 120 mg/kg. Finally, Bagheri et al. observed good efficacy with a cumulative dose of 102.5 mg/kg. These findings provide valuable insights into the various dosing strategies and their outcomes in treating PDA in preterm infants.[12]

Our review highlights that the total dose for a complete intravenous course of paracetamol, as reported in all twelve RCTs, ranges from 102.5 mg/kg to 180 mg/kg.[12] Interestingly, the efficacy of paracetamol remains similar across most of these dosage ranges, suggesting that there may not be any advantage in administering higher dosage regimens. The majority of clinical trials on the use of paracetamol have relied on trial-and-error methods of drug dosage and serum levels rather than trying to predict the kinetic behaviour of paracetamol through comprehensive pharmacokinetic modelling.

Hammerman et al.'s chance discovery (2011) of using oral paracetamol to close PDA has led to several intravenous trials using the same oral dose (15mg/kg).[26] However, no pharmacokinetic or pharmacodynamic studies on IV paracetamol in preterm infants have supported this use.[21] Reports suggest that higher doses of paracetamol can cause toxicity. In a retrospective study, Tekgündüz et al. reported that they encountered elevated transaminase levels at doses of IV paracetamol of 15 mg/kg every 6 hours (cumulative dose 180 mg/kg/day).[27] Terrin et al. recommended a limit of 45 mg/kg per day to avoid potential toxicity while maintaining similar efficacy rates.[28]

Intravenous paracetamol is not licensed for use in premature infants < 32 weeks' gestation (vide BNFc) in the UK.[18] While there have been several observational studies and RCTs that have used intravenous paracetamol for ductal closure in premature infants, the doses of IV paracetamol used in these trials were based on empirical evidence, as there were no available data regarding the pharmacokinetics (PK) and pharmacodynamics of paracetamol use in preterm neonates for this potential new indication.

Allegaert et al. conducted one of the most comprehensive studies on the pharmacokinetic evidence of IV paracetamol use in premature neonates.[20,22] The authors demonstrated that an increased volume of distribution supports the use of a loading dose in neonates. They also reported that size (described by patient weight) is the major co-variate contributing to paracetamol clearance variance in neonates, as paracetamol clearance (mg/kg/hour) increases marginally throughout the neonatal

life.[20,22,23] The authors suggested a loading dose of 20 mg/kg followed by 10 mg/kg every 6 hours within the age range evaluated (32-44 weeks gestational age) to achieve the desired mean concentration of the drug.

In vivo, the serum concentration of a drug at any given time is in a state of continuous dynamic equilibrium with its absorption, distribution, tissue storage, liberation and elimination. Hence, any evaluation of the serum therapeutic concentration requires comprehensive pharmacokinetic compartment modelling. Compartment models in pharmacokinetic studies simulate the dynamic interplays of drug absorption, distribution, and elimination. A single-compartment linear disposition pharmacokinetic model (zero-order input, first-order elimination) is supposedly the least accurate evaluation, as it assumes a homogeneous distribution of the drug throughout the body. The pharmacokinetic evaluation by Allegaert et al. is based on a two-compartment model, which is better than a single-compartment model. [20,22]

In addition, factors like renal disease, liver impairment, and hyperbilirubinemia can all affect paracetamol metabolism and clearance. Any infant on enzyme-inducing antiepileptic drugs, including phenobarbitone, rifampicin, or phenytoin, should be monitored closely because these drugs can potentially interact with paracetamol.

At this point, it appears that there will be definite advantages to considering the continuous infusion of paracetamol rather than bolus administration to achieve a steady-state serum concentration of paracetamol. In a recent study by Cakir et al., the authors compared continuous infusion vs standard intermittent bolus infusion of paracetamol to treat PDA.[25] Despite the apparent theoretical advantage of continuous infusion in achieving consistent therapeutic serum concentration, the study did not find any merit in the use of paracetamol infusion in comparison to intermittent bolus doses for the pharmacological treatment of PDA. On the contrary, lower rates of PDA-related morbidity, such as BPD, NEC, and the need for PDA ligation, were noted in the intermittent dosing arm.

Following these observations and in keeping with the pharmacokinetic study by Allegaert et al., a loading dose of 20 mg/kg/day was proposed, followed by 10 mg/kg/day every six hours for three days, for the PAIR trial. This provides safety in a relatively low dose (cumulative dose 130 mg/kg/day) without compromising efficacy. This dosage regimen has unanimous consensus in our NICU and is also recommended by the Northwest Operational Delivery Network (NWODN), UK, for managing hsPDA in preterm infants. This NWODN consensus guideline applies across the entire North-West region of the UK, including North Wales, Manchester, and Liverpool tertiary neonatal units.[19] It has been incorporated into our clinical practice, so if infants did not participate in our trial and the clinicians felt the need to use IV paracetamol, they will receive this dose for the treatment of hsPDA. In summary, based on the evidence in the literature, the dose suggested in the PAIR trial is likely to provide the best balance of efficacy with no reported side effects. As the PAIR trial was set up, new evidence on paracetamol dosing in premature infants came up. A French group of researchers (Bouazza et al. 2021) conducted a population-based pharmacokinetic single-compartment model (using Monolix[R] software) for paracetamol use in treating hsPDA in premature infants. This initiative was taken as there was no convincing data on the pharmacokinetic properties of paracetamol for treating hsPDA in premature infants. Their study demonstrated that the dosages used (first day 42.5 mg/kg, then 30 mg/kg/day) allowed for the maximum inhibition response from paracetamol regarding the time to close the ductus. It was shown that the ductal constriction rate as a response to paracetamol administration plateaus after the third day of treatment, as plotted against time (Figure 2). However, this study also pointed out a reduced efficacy of paracetamol in extremely preterm neonates (below 27 weeks). Therefore, they suggested dose-finding research focusing specifically on extremely preterm neonates (<26 weeks of gestation) to evaluate treatment efficacy and toxicity.[21]

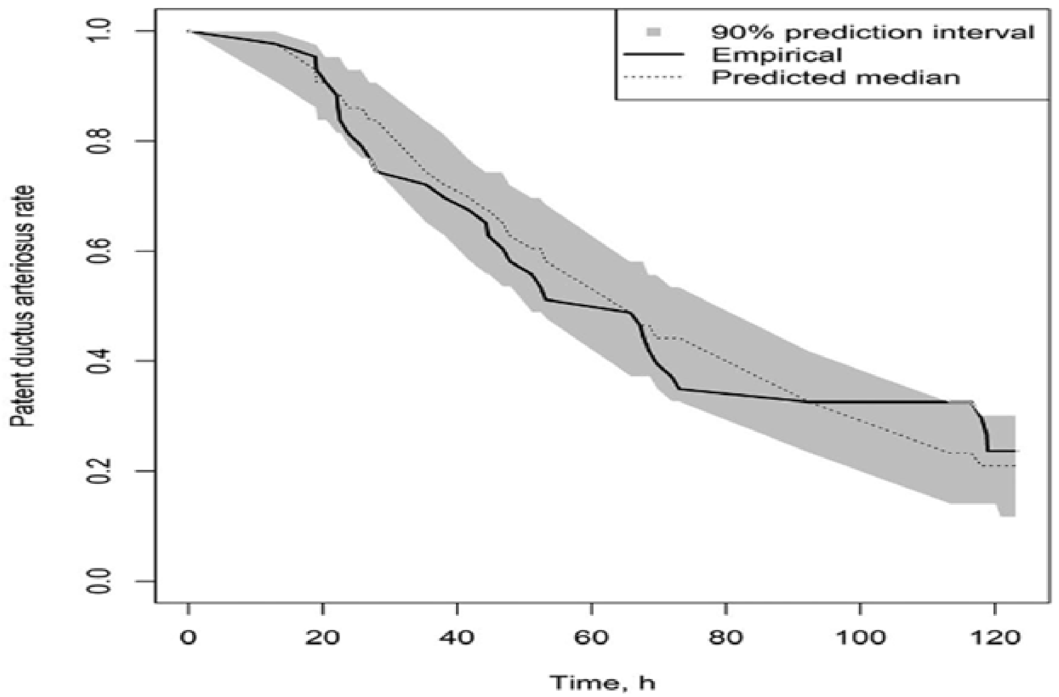


**Figure 2: Dose-Response Graph of IV Paracetamol**

*The figure demonstrates the relationship between the PDA closure rate and the treatment duration beyond 72 hours, where the ductal constriction response plateaus.*

**Table 2: Summary of PK and PD from RCTs using IV Paracetamol for PDA management [12]**

| Study | Pharmacokinetics | Pharmacodynamics |
|---|---|---|

| Authors | Year/ Country/ Intervention | Sample size | Eligible population (Gestation) | Criteria for inclusion | Regime | Total dose in mg/kg | Drug levels? | Therapeutic efficacy & Statistical significance | Side Effects of Paracetamol (PCM) |
|---|---|---|---|---|---|---|---|---|---|
| Harkin et al. | 2016/ Finland<br>PCM^ (n=23)<br>Vs.*<br>Placebo (n=25) | 48 | <32 weeks | Echo only | A loading dose of 20mg/kg followed by 7.5mg/kg 6 hourly for 4 days | 133.5 | Not performed | Early closure of PDA (177 hours versus 338 hours). Statistically significant against placebo | No detectable adverse effects with PCM |
| El-Mashad et al. | 2017/ Egypt<br>PCM (n=100)<br>Vs. Ibuprofen (n=100)<br>Vs. Indomethacin (n=100) | 300 | <28 weeks/ <1500 grams | Clinical + echo | 15mg/kg every 6 hours for 3 days | 180 | Not performed | 80% PCM efficacy versus 77% ibuprofen and 81% indomethacin. No significant difference in adverse effects. | Significant increase in creatinine and BUN in the Ibuprofen and Indomethacin groups along with oliguria and thrombocytopenia. No such adverse effect was observed in the PCM-treated group |
| Bagheri et al. | 2018/ Iran<br>PCM (n=80)<br>Vs.<br>Placebo (n=80) | 160 | ≤ 34 weeks | Clinical examination only to rule out CHD~ | 20mg/kg loading followed by 7.5mg/kg every 6 hours for 3 days | 102.5 | Not performed | In the PCM group, 15% of PDA remained open, while in the placebo group, 71.25% remained open. Not statistically significant. | No hepatic complications occurred during the hospital stay |
| Hochwald et al. | 2018/ Israel<br>PCM+ Ibuprofen (n=12)<br>Vs.<br>Placebo+ Ibuprofen (n=12) | 24 | <32 weeks | Clinical + echo | A loading dose of 20 mg /kg followed by 10mg/kg 6 hours for 3 days (a total of 12 doses) ** | 130 | Not performed | 83% in the PCM-added group Vs. 42% in the placebo-added group. Not statistically significant | No adverse clinical or laboratory effects were noted in the PCM-added group |
| Ghaderian et al. | 2019/ Iran<br>PCM (n=20)<br>Vs.<br>Ibuprofen (n=20) | 40 | <32 weeks<br><1500 grams | | 15mg/kg every 6 hours for 2 days | 120 | Not performed | 60% with PCM Vs. 65% with ibuprofen after the first course | No significant difference in adverse effects |
| Jafari et al. | 2019/ Iran<br>PCM (n=15)<br>Vs.<br>Ibuprofen (n=15) | 30 | 28-34 weeks | Echo only within 72 hours (early treatment) | 15mg/kg every 6 hours for 3 days | 180 | Not performed | PDA closure rates with PCM 87.5% Vs. 92.1% with ibuprofen | No comments on side effects. The study had different secondary outcomes |
| Dani et al. | 2020/ Italy<br>PCM (n=58)<br>Vs.<br>Ibuprofen (n=52) | 110 | <32 weeks | Clinical + echo | 15mg/kg every 6 hours for 3 days | 180 | Not performed | PCM 52% closure rate Vs. ibuprofen 78%. Success rates in constriction were similar. Not significant. | No difference in adverse effects between the two groups |

| | | | | | | | | | |
|---|---|---|---|---|---|---|---|---|---|
| Meena et al. | 2020/ India IV PCM (n=35) Vs. PO$ ibuprofen (n=35) Vs. PO indomethacin n (n=35) | 105 | <37 weeks | Clinical + echo | 15 mg/kg 6 hourly for 3 days | 180 | Not performed | Cumulative closure rate 71.43% PCM vs 77.14% in ibuprofen group vs 68% in indomethacin group. Not significant. | IV PCM had a better safety profile. Serum creatinine and BUN were significantly raised in the indomethacin and ibuprofen groups |
| Tauber et al. | 2020/US IV PCM (n=5) Vs. IV Ibuprofen (n=5) | 10 | <30 weeks | Echo only in the first 2 weeks of life | 15mg/kg every 6 hours for 3 days | 180 | Not performed | All infants in the ibuprofen group and 2/5 infants in the PCM group needed subsequent pharmacologic treatment. | No side effects were reported in either group |
| Schindler et al. | 2021/Aussie PCM (n=29) Vs. Placebo (n=29) | 58 | <29 weeks | Echo only | 15mg/kg loading followed by 7.5mg every 6 hours for 5 days | 157.5 | Not performed | The PCM group required PDA intervention, 21% vs 59% in the placebo group. Statistically significant | No adverse effects were reported in the PCM group |
| Davidson et al. | 2021/US IV PCM (n=17) Vs. IV indomethacin (n=20) | 37 | ≤32 weeks or ≤1500 grams | Echo only | 15 mg/kg 6 hourly for 3 days | 180 | Not performed | One (5.9%) hsPDA in the PCM group had successful treatment compared to 11 (55%) in the indomethacin group | The side effects of both drugs were similar |
| Shahmirzadi et al. | 2021/Iran IV PCM (n=23) Vs. PO Ibuprofen (n=17) | 40 | Premature infants (gestation unknown) | Echo diagnosis of PDA | 15 mg/kg every 6 hours for 2 days | 120 | Not performed | No statistically significant relationship in PDA closure between the treatment groups | Incidence and severity of GI bleeding, feed intolerance and NEC were significantly higher in infants receiving PCM. Statistically significant. No difference in biochemical parameters |

*^PCM Paracetamol, *Vs. versus, ~CHD Congenital Heart Disease, $PO per-oral **Same dose regimen as PAIR trial*

**Bibliography:**


1. Noureldein M, Hu K, Groucutt J, Heaver R, Gurusamy K. Paracetamol for patent ductus arteriosus in preterm infants: a UK national survey. The Journal of Maternal-Fetal & Neonatal Medicine. 2022;35(7):1408-11.
2. Mukherjee A, Jadhav V, Gupta A. Off-label use of paracetamol in managing patent ductus arteriosus across neonatal intensive care units in the UK. Archives of Disease in Childhood-Fetal and Neonatal Edition. 2020.
3. Gupta A, Mukherjee A. Do we know how to treat PDA with paracetamol? Current evidence on the pharmacokinetics and pharmacodynamics of paracetamol for hsPDA closure in extreme preterm infants. Infant; 2021.
4. El-Mashad AE-R, El-Mahdy H, El Amrousy D, Elgendy M. Comparative study of the efficacy and safety of paracetamol, ibuprofen, and indomethacin in closure of patent ductus arteriosus in preterm neonates. European journal of pediatrics. 2017; 176:233-40.
5. Jafari N, Mahdian Jouibari R, Ebadi A, Kamali K, Abdolahzadeh S, Hosseini M. A Comparison between the safety and efficacy of IV paracetamol (Acetaminophen) and IV Ibuprofen in treating premature neonates with patent ductus arteriosus. Journal of Iranian Medical Council. 2019;2(4):66-73.
6. Dani C, Lista G, Bianchi S, Mosca F, Schena F, Ramenghi L, et al. Intravenous paracetamol in comparison with ibuprofen for the treatment of patent ductus arteriosus in preterm infants: a randomized controlled trial. European Journal of Pediatrics. 2021;180(3):807-16.
7. Tauber KA, King R, Colon M. Intravenous acetaminophen vs intravenous ibuprofen to close a patent ductus arteriosus closure: a pilot randomized controlled trial. Health Science Reports. 2020;3(3).
8. Varvarigou A, Bardin CL, Beharry K, Chemtob S, Papageorgiou A, Aranda JV. Early ibuprofen administration to prevent patent ductus arteriosus in premature newborn infants. Jama. 1996;275(7):539-44.
9. Su B-H, Lin H-C, Chiu H-Y, Hsieh H-Y, Chen H-H, Tsai Y-C. Comparison of ibuprofen and indometacin for early-targeted treatment of patent ductus arteriosus in extremely premature infants: a randomised controlled trial. Archives of Disease in Childhood-Fetal and Neonatal Edition. 2008;93(2): F94-F9.
10. Jain A, Shah PS. Diagnosis, evaluation, and management of patent ductus arteriosus in preterm neonates. JAMA pediatrics. 2015;169(9):863-72.
11. Pereira S. Comparison of PDA diameter measurements using color and 2D echocardiography in newborn infants. Journal of Neonatal-Perinatal Medicine. 2023;16(1):137-40.
12. Katsaras DN, Katsaras GN, Chatziravdeli VI, Papavasileiou GN, Touloupaki M, Mitsiakos G, et al. Comparative safety and efficacy of paracetamol versus non-steroidal anti-inflammatory agents in neonates with patent ductus arteriosus: A systematic review and meta-analysis of randomized controlled trials. British Journal of Clinical Pharmacology. 2022;88(7):3078-100.
13. Semberova J, Sirc J, Miletin J, Kucera J, Berka I, Sebkova S, et al. Spontaneous closure of patent ductus arteriosus in infants≤ 1500 g. Pediatrics. 2017;140(2).
14. De Klerk JC, Engbers AG, Van Beek F, Flint RB, Reiss IK, Völler S, et al. Spontaneous closure of the ductus arteriosus in preterm infants: a systematic review. Frontiers in Pediatrics. 2020; 8:541.
15. Sellmer A, Bjerre JV, Schmidt MR, McNamara PJ, Hjortdal VE, Høst B, et al. Morbidity and mortality in preterm neonates with patent ductus arteriosus on day 3. Archives of Disease in Childhood-Fetal and Neonatal Edition. 2013;98(6): F505-F10.
16. Kluckow M, Carlisle H, Broom M, Woods P, Jeffery M, Desai D, et al. A pilot randomised blinded placebo-controlled trial of paracetamol for later treatment of a patent ductus arteriosus. Journal of Perinatology. 2019;39(1):102-7.

17. Xiao Y, Liu H, Hu R, You Q, Zeng M, Jiang X. Efficacy and safety of paracetamol for patent ductus arteriosus closure in preterm infants: an updated systematic review and meta-analysis. Frontiers in Pediatrics. 2020; 7:568.
18. BNFc, UK.

19. NWODN [updated 07/09/2023; cited 2023 07/09/2023]. Available from: https://www.neonatalnetwork.co.uk/nwnodn/wp-content/uploads/2023/09/GL-ODN-09-NW-Guideline-for-the-Management-of-PDA-Revised.pdf.
20. Allegaert K, Van der Marel CD, Debeer A, Pluim M, Van Lingen R, Vanhole C, et al. Pharmacokinetics of single dose intravenous propacetamol in neonates: effect of gestational age. Archives of Disease in Childhood-Fetal and Neonatal Edition. 2004;89(1): F25-F8.
21. Bouazza N, Treluyer JM, Foissac F, Urien S, Aikio O, Roze JC, et al. Pharmacokinetics of intravenous paracetamol (acetaminophen) and ductus arteriosus closure after premature birth. Clinical Pharmacology & Therapeutics. 2021;110(4):1087-95.
22. Allegaert K, Palmer GM, Anderson BJ. The pharmacokinetics of intravenous paracetamol in neonates: size matters most. Archives of disease in childhood. 2011;96(6):575-80.
23. Palmer G, Atkins M, Anderson B, Smith K, Culnane T, McNally C, et al. IV acetaminophen pharmacokinetics in neonates after multiple doses. British journal of anaesthesia. 2008;101(4):523-30.

24. van der Horst J, Manville RW, Hayes K, Thomsen MB, Abbott GW, Jepps TA. Acetaminophen (paracetamol) metabolites induce vasodilation and hypotension by activating Kv7 potassium channels directly and indirectly. Arteriosclerosis, thrombosis, and vascular biology. 2020;40(5):1207-19.
25. Cakir U, Tayman C, Karacaglar NB, Beser E, Ceran B, Unsal H. Comparison of the effect of continuous and standard intermittent bolus paracetamol infusion on patent ductus arteriosus. European Journal of Pediatrics. 2021; 180:433-40.
26. Hammerman C, Bin-Nun A, Markovitch E, Schimmel MS, Kaplan M, Fink D. Ductal closure with paracetamol: a surprising new approach to patent ductus arteriosus treatment. Pediatrics. 2011;128(6): e1618-e21.

27. Tekgündüz KŞ, Ceviz N, Caner İ, Olgun H, Demirelli Y, Yolcu C, et al. Intravenous paracetamol with a lower dose is also effective for the treatment of patent ductus arteriosus in pre-term infants. Cardiology in the Young. 2015;25(6):1060-4.
28. Terrin G, Conte F, Scipione A, Bacchio E, Conti MG, Ferro R, et al. Efficacy of paracetamol for the treatment of patent ductus arteriosus in preterm neonates. Italian journal of pediatrics. 2014; 40:1-4.
